# Transcriptomic analysis of Dubas bug (*Ommatissus lybicus* Bergevin) infestation to Date Palm

**DOI:** 10.1038/s41598-020-67438-z

**Published:** 2020-07-13

**Authors:** Abdul Latif Khan, Sajjad Asaf, Adil Khan, Arif Khan, Muhammad Imran, Ahmed Al-Harrasi, In-Jung Lee, Ahmed Al-Rawahi

**Affiliations:** 1 Natural and Medical Sciences Research Center, University of Nizwa, 616, Nizwa, Oman; 2 Genomics Group, Faculty of Biosciences and Aquaculture, Nord University, 8049 Bodø, Norway; 3 School of Applied Biosciences, Kyungpook National University, Daegu, South Korea

**Keywords:** Pattern recognition receptors in plants, Plant signalling, Herbivory

## Abstract

Date palm (*Phoenix dactylifera* L.) and its fruit possess sociocultural, health and economic importance in Middle East. The date palm plantations are prone to Dubas bug (DB; *Ommatissus lybicus* DeBergevin; Homoptera: Tropiduchidae) attacks that severely damages the tree’s growth and reduces fruit production. However, the transcriptome related datasets are not known to understand how DB activates physiological and gene regulatory mechanisms during infestation. Hence, we performed RNA-Seq of leaf infected with or without DB to understand the molecular responses of date palm seedlings. Before doing that, we noticed that DB infestation significantly increase superoxide anion and malondialdehyde production to two-folds as compared to healthy control. Stress-responsive genes such as *proline transporter 2*, *NADP-dependent glyceraldehyde* and *superoxide dismutase* were found significantly upregulated in infected seedlings. The infection repercussions were also revealed by significantly higher contents of endogenous phytohormonal signaling of jasmonic acid (JA) and salicylic acid (SA) compared with control. These findings persuaded to dig out intrinsic mechanisms and gene regulatory networks behind DB infestation to date palm by RNA-Seq analysis. Transcriptome analysis revealed upregulation of 6,919 genes and down-regulation of 2,695 genes in leaf during the infection process. The differentially expressed genes were mostly belongs to cellular functions (calcium and MAPK), phytohormones (auxin, gibberellins, abscisic acid, JA and SA), and secondary metabolites (especially coumarinates and gossypol). The data showed that defense responses were aggravated by gene networks involved in hypersensitive responses (*PAR1, RIN4, PBS1* etc.). In conclusion, the results revealed that date palm’s leaf up-regulates both cellular and phytohormonal determinants, followed by intrinsic hypersensitive responses to counter infestation process by Dubas bug.

## Introduction

Date palm (*Phoenix dactylifera* L.) is one of the oldest fruits crop and has played particularly important role in the culture, economy and well-being of the people of Arabian region^1^. It is widely grown in arid and semi-arid region , and distributed across 24 countries^2^. The fruit is an important part of dietary intake due to its significant nutritional values. Like other countries in Arabian Peninsula, there are more than 300 date palm cultivars in Oman—the 8th largest producers of date fruits. Although with improved breading and tissue culture technologies, highly resistant varieties are cultivated in oasis, however, still the tree is confronted with pathogenic and insect attacks, hence reducing its growth, yield and production^3,4^. The literature shows that date palm fruit decline significantly due to the attack of Dubas bug (*Ommatissus lybicus* Bergevin, Homoptera: Tropiduchidae) in the Middle East and North Africa, which is considered a major pests^3–5^.

Dubas bug (DB) was identified by Blumberg for the first time in the Tigris-Euphrates River Valley. Later on, he claimed that DB spread from its primary origin to other regions^3,6,7^. During active period, Dubas bug nymphs hatch and feed on the nutrient sap of the leaf^3,6^. Nymphs pass through five growth instars^8,9^, with adult female DB grows to 5–6 mm and males to 3–3.5 mm in length^10,11^. Two populations of DB are produced each year. The summer generation of nymph’s hatch in mid to late April. While feeding, the insect produces excreta in the form of honeydew on the leaflets and accumulates on top of the leaf—a shining droplet full of sugar and other constituents. This become the onset of mainstay problem by development of pathogenic infection (black sooty mold on the foliage), further damaging the leaf parts via chlorosis^12^. This consequently cause reduction in the photosynthetic rates^9,13^. Prolonged and high intensity of infestation results in the flagging and destruction of palm plantations^14^. Thus, overall there are various factors that influence the infestation patterns, however, this needs detailed in-depth molecular approaches ensure proficient datasets for further studies.

Dealing with DBs infestations, various approaches have focused on the use of insecticides. In Iraq and Israel directly inject dichlorvos (DDVP), and systemic carbamates (e.g., aldicarb and butocarboxim) respectively, into infected palms that are found successful in controlling infestations^3,15^. However, these strategies are consider harmful for environment, human and to other species e.g., *Oligosita* sp. (Hymenoptera: Trichogammidae, *Aprostocetus* sp. (Hymenoptera: Eulophidae), and *Runcinia* sp. (Aranae: Thomsidae)^3^. In addition to that, studies have shown that after application of insecticides, some pesticide residues remain on the date palm fruits for up to sixty^16^. Although some recent work has been carried to understand the DB infestation and life cycle, however, how the date palm responds to the infection process has not been well understood. Elucidating such infestation based genetic responses by the host itself will help to explain the innate immunity mechanism against prolong infections and to give alternatives and specific targets for date palm breeder in developing resistant cultivars.

Although fungal infestation and host physio-molecular responses have been well studied in various crop plants, however, studies related to arid land date palm has been frequently overlooked. Broadly, insect attack on leaf is preset of wounding or injury to the tissue, also in case of DB, which follows with the fungal infection—a duo synergistic action triggering a race for feed and reproduction^17^. This initiate activation of defense related mechanisms such as production of antioxidants and signaling cascades of endogenous phytohormones such as jasmonic acid and salicylic acid, whereas some trees tends to produce volatile and resinous components to counteracts such attacks^17–22^. However, such responses could vary among different species whereas it depends on insect infestation mode and intensity. Particularly, the way DB attack might be similar to other insect; however, the post-infection process is restricted only to the species.

**Figure 1:**
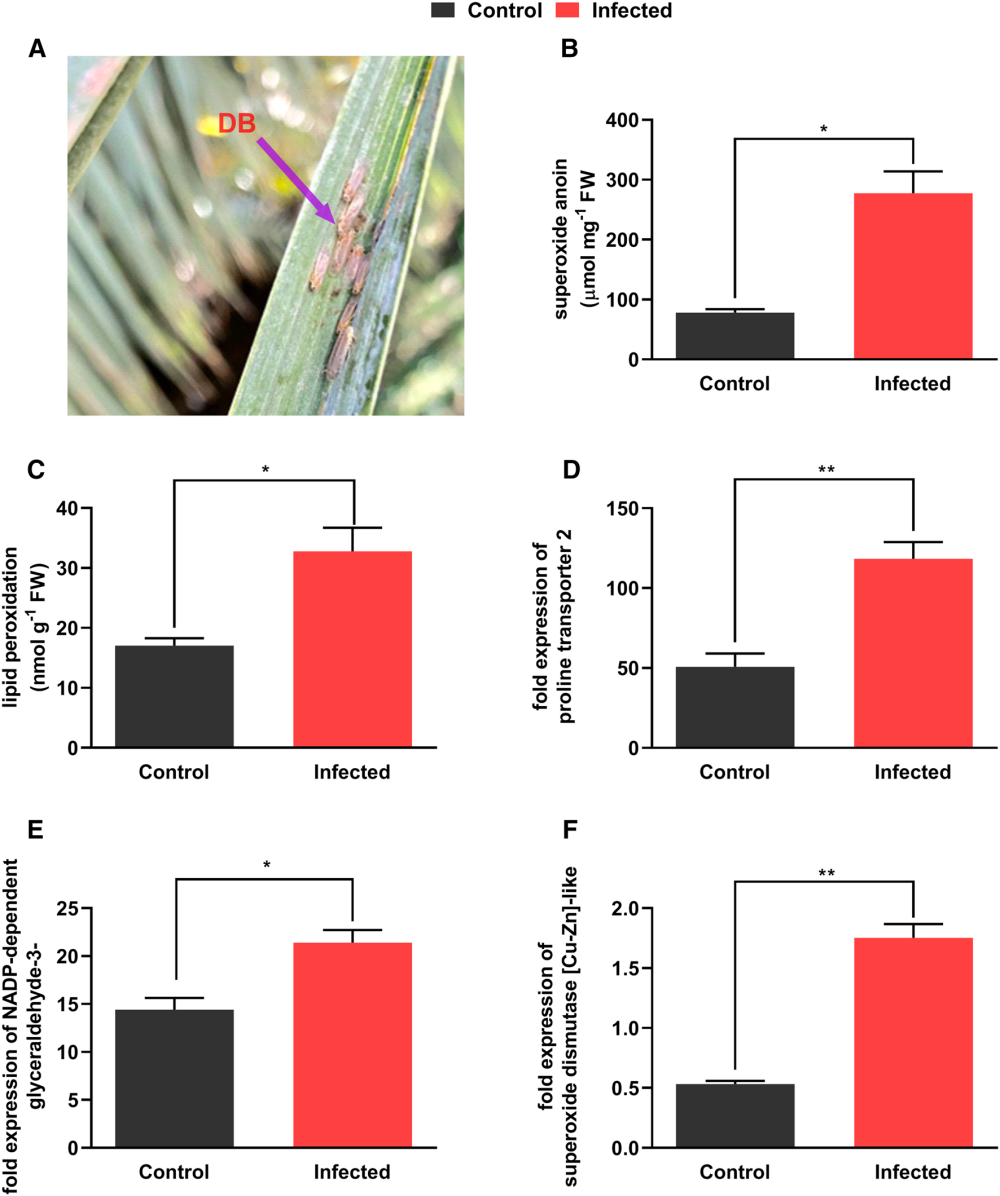
Date palm leaves infected by Dubas bug (DB) (**A**), superoxide anion (**B**), measurements of MDA content (**C**), fold change expression of Proline transporter 2 (**D**), fold change expression of NADP-dependent glyceraldehyde-3- (**E**), fold change expression of superoxide dismutase [Cu–Zn]-like (**F**). *, **, indicate a significant difference between healthy and infected sample where *p* ˂ 0.05, and 0.01 respectively.

In case of date palm tree, there are a few previous studies^23–26^ explaining the physiology and genomics, however, very few studies have also shown the differential gene expression of date palm during abiotic stress conditions^27^. There are recent studies performed to understand the infestation process and related gene’s regulation in aphid feedings to susceptible plants^28^, *Ostinia furnacalis* leaf feeding to maize^29,30^, soldier fly on sugarcanes in Australia, aphid attacks on wheat crops, and Lepidoptera species infection to cotton^31,32^. These studies have used detailed RNA-Seq based method to point out various underlying mechanisms during herbivory infection. Such studies utilizing the ‘omics-based approaches could help in finding out resistance and attack mechanisms that could broadly improve the control of infection strategies. Contrarily, there are no studies till date on the transcriptomic analysis of DB infection to date palm. Hence, in current study, we aimed to understand the underlying mechanisms of DB infection on the leaf of date palm. For this purpose, healthy control and infected date palm samples were assessed initially for their responses against oxidative stress and regulation of endogenous phytohormonal during DB attack (Fig. 1A). These intriguing results persuaded further to perform in-depth next-generation sequencing (RNA-Seq) approaches to identify and elucidate the gene expression network(s) during infection process. This study was performed for the first time to usher the gene expression patterns, activation of defense related pathways, triggering of endogenous phytohormones and generating transcript datasets for future studies during DB attack to date palm. This will address how the date palm respond to DB infection and what molecular pathways are activated during defense mechanisms.

## Results

### Oxidative stress and gene expression during Dubas bug infection process

To understand the level of effects in stress inception on date palm leaf under DB infection, initial assessments were made by analyzing several key biochemical and molecular determinants. The results showed that DB infestation significantly increased (*p* < 0.001; two folds) superoxide anion (O_2_^−^) as compared to healthy control (Fig. 1B). Lipid peroxidation, a stress indicative process during biotic and abiotic stresses, showed that malondialdehyde (MDA; a bi-product of lipid peroxidation) was significantly (*p* < 0.001; two and half fold) higher in infected leaf as compared to healthy (Fig. 1C). We noted a significantly higher (*p* < 0.001; 83%) amount of superoxide dismutase in infected plants as compared to control. Hence, the results reveal that DB infection predominantly cause severe damages to the leaf tissues whereas date palm in turn intercept stress factors by activating antioxidant apparatus. Similarly, the photosynthetic pigments including Chl *a*, *b,* Chl *a* + *b* and carotenoid (Car) decreased significantly (17.02, 23.89, 18.78 and 13.85% respectively) in DB infected (Figure S1A–D). Whilst, phenolic acid increased significantly in DB infected leaf tissues (Figure S1E). However, polyphenol level did not change significantly in infected leaves as compared to healthy (Figure S1F).

In molecular determinants, protective osmolytes such as proline are activated to protect cellular osmotic balance, cell-wall modification and synthesis^33^ and the results of proline transporter 2 gene expression was significantly (*p* < 0.05) up-regulated in DB infected leaves as compared to control (Fig. 1D). Whereas, *NADP-dependent glyceraldehyde-3-phosphate dehydrogenase* was significantly (*p* < 0.001) up-regulated during DB infection (Fig. 1E) and was previously shown to mobilize ascorbate–glutathione pathway and NADPH-dependent thioredoxin reductase during apoplastic oxidative burst during biotic stresses^34^. A similar perspective was also noted for SOD synthase related gene with exponentially significant activation in DB infection in date palm leaves as compared to control (Fig. 1F). The *abscisic acid receptor PYL4-Like* was significantly (*p* < 0.02) upregulated than control (Figure S2), indicating higher stress incursion in infected leaf.

### Endogenous phytohormonal regulation

**Figure 2:**
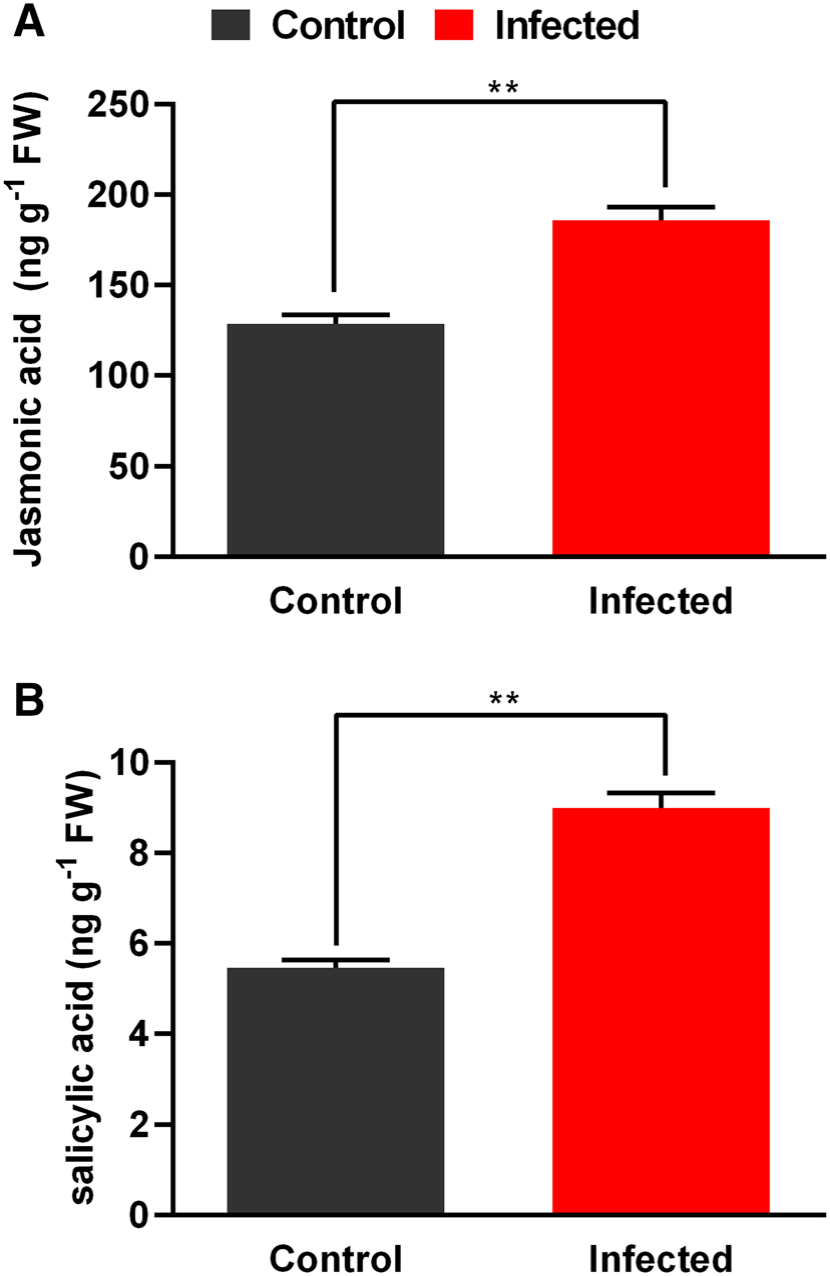
Levels of stress related hormone in healthy and infected date palm tree. Measurement of Jasmonic acid (**A**), measurements of salicylic acid (**B**). **, indicate a significant difference between healthy and infected sample where *p* ˂ 0.01.

To counteract the negative impact of herbivory, endogenous phytohormones such as jasmonic acid (JA) and salicylic acid (SA) have been known signaling players in defense responses^17^. We found that JA was significantly (*p* < 0.001) higher (double-fold; 90%) in DB infected date palm leaves as compared to control (Fig. 2A). SA was also significantly higher (*p* < 0.001; one and half fold) during the infection process (Fig. 2B). However, the amount of SA synthesized was extremely negligible as compared to JA, suggesting a more potent role in DB infestation process^35^.

### Transcriptome sequencing, assembly and annotation

Persuaded by significant biochemical and gene expression results, further in-depth next-generation sequencing (RNA-Seq) approaches were used to elucidate the intricate gene expression networks during infection process. Using standard protocols of RNA-Seq, we generated total of 92 million reads from healthy and DB infected date palm leaves. For downstream gene expression analysis, after filtration only high-quality reads (ranging from 1,600 to 1,900 MB) were mapped to the date palm (DPV01 pdS000001) genome. The sequences of healthy and infected sample were analyzed for differential gene expression (DEGs; Figure S3; Supplementary dataset 1). Among 10,042 DEGs, 6,919 and 2,695 were up and down-regulated genes respectively (Supplementary dataset 1; Figure S3). A total of 140 genes were deferentially expressed in infected and control of these 31 were up-regulated and 109 were down-regulated (Figure S3). In the healthy leaves, although many genes have a log fold ratio higher than 1, but these were non-significant FDR. In the control/infected comparison a considerable portion of the analyzed genes were significantly (*p* < 0.05) regulated (Figure S3).

### Gene ontology (GO) classification and functional annotation

**Figure 3:**
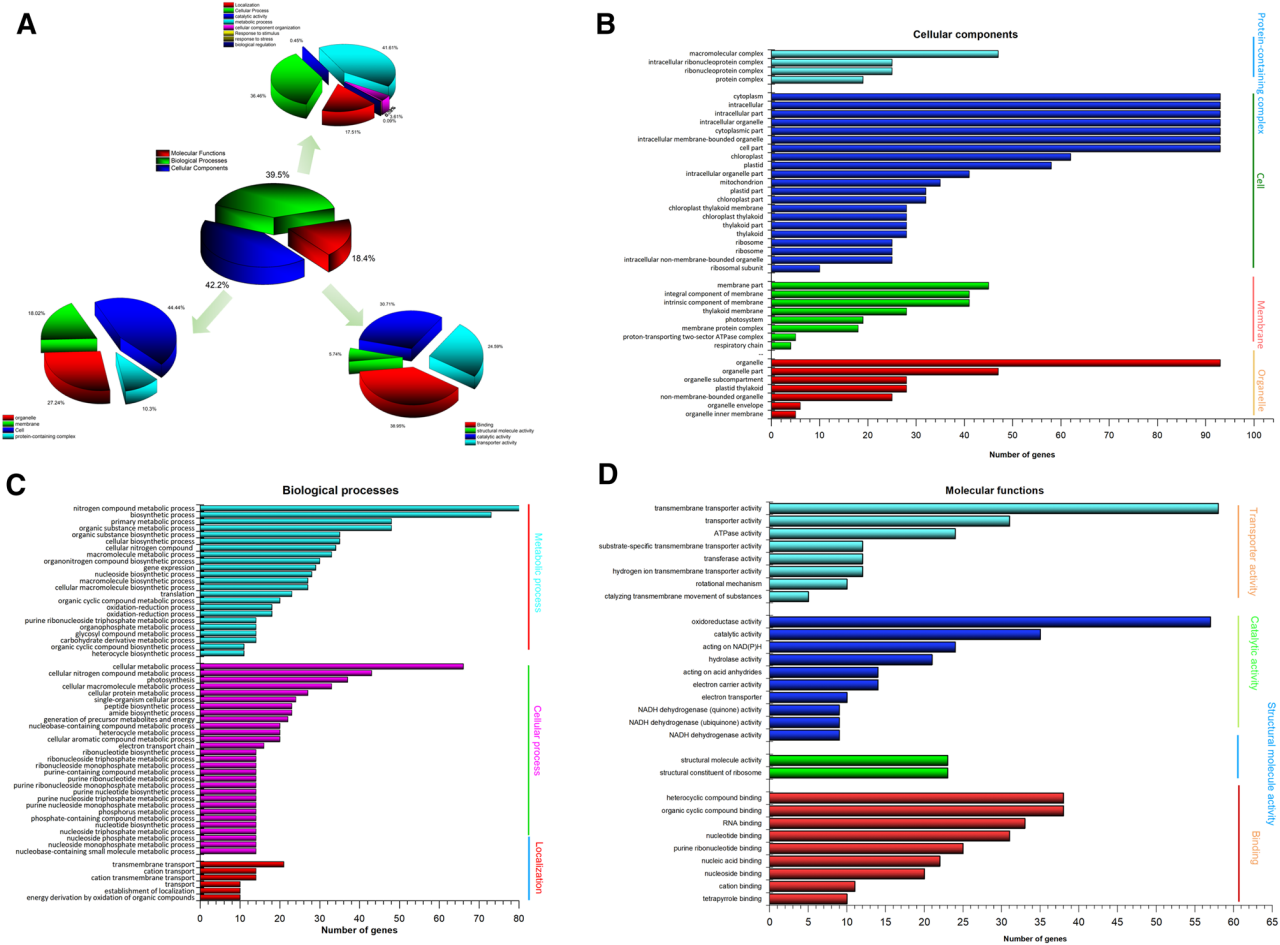
(**A**) Assembled DEGs were functionally classified by Gene Ontology categorization. The DEGs corresponded to three main categories: biological process, cellular component, and molecular Function, (**B**) gene Ontology (GO) annotation of differentially expressed genes (DEGs) associated to Cellular components, (**C**) gene Ontology (GO) annotation of differentially expressed genes (DEGs) associated to Biological processes, (**D**) gene Ontology (GO) annotation of differentially expressed genes (DEGs) associated to Molecular functions. The x-axis shows the number of genes while the y-axis shows gene function annotation of GO categories.

The results of GO functionality showed approximately 4,341 Differentially Expressed Genes (DEGs) that were classified into cellular component (CC), biological process (BP) and molecular function (MF). Of these, about 797 (18.4%), 1,713 (39.5%) and 1,831 (42.2%) DEGs were associated with MF, BP and CC respectively (Fig. 3A). The cellular processes mainly included cellular growth 535 DEGs (44.4%), membrane 217 DEGs (18.02%), organelle 328 DEGs (27.24%), and protein-containing complex 124 DEGs (10.3%). Of these 1,713 BP associated DEGs classified, most were associated with metabolic process 461 DEGs, (41.61%), cellular process 404, (36.46%), localization 194, (17.5%), cellular component organization 40 DEGs (3.61%) aspects of biological processes. Detail GO term analysis and deferentially expressed DEGs associated with MF, CC and BP are showed in Fig. 3A–D. Similarly, about 312 DEGs (18.4%) were associated with Binding, 246 DEGs (38.95%) with catalytic activity and 197 DEGs (30.71%) with transporter activity out of 797 DEGs associated with molecular functions (Fig. 3D).

### Calcium and MAPK signaling cascades during Dubas bug interactions

**Figure 4:**
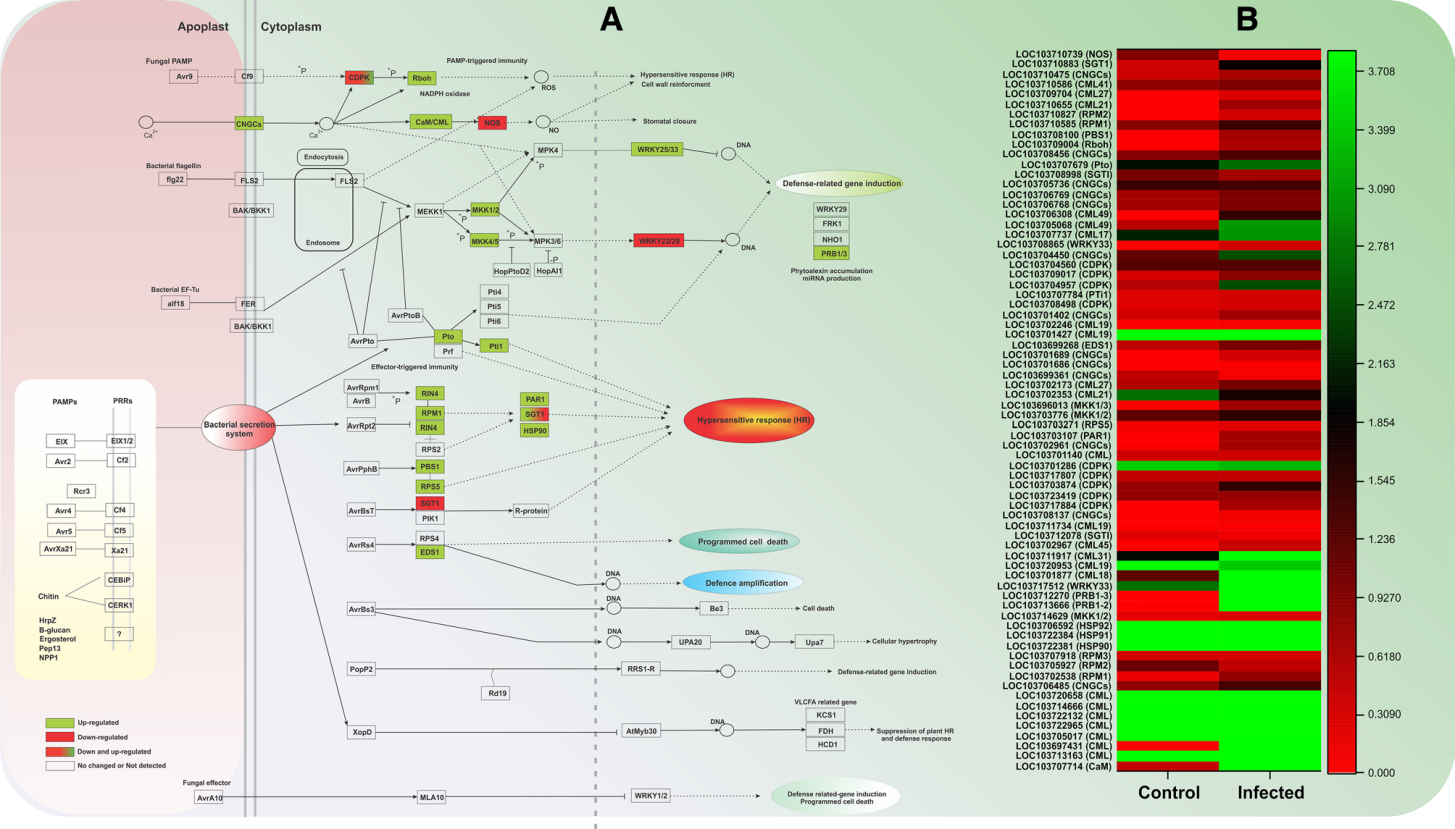
(**A**) DEGs involved in plant -pathogen interaction pathway in date palm infected Dubas bug based on KEGG analysis, (**B**) expression patterns of representative DEGs in plant -pathogen interaction pathway.

According to KEGG pathways analysis, about 73 DEGs were found related to plant-pathogen interaction pathways, among that 17 were commonly deferentially expressed across the time points (Fig. 4A; Supplementary dataset 2). About 64 DEGs were found up-regulated while 13 DEGs were found down-regulated (Fig. 4A). Cytoplasmic Ca^2+^ precipitously aggregates during the perception of pathogen associated molecular patterns (PAMPs). We revealed that cyclicnucleotide-gated channels (CNGCs), Rboh and CaM/CML have been up regulated while CDPK is found up as well as down-regulated after DB infection. Similarly, the LOC103717884, LOC103723419, LOC103703874, LOC103701286 and LOC10371780 were found related to calcium dependent protein kinase (CDPK) family. CDPKs, on the other hand, are phosphorylated to reciprocate the hyper-sensitive response (HR). Similarly, calcium related locus (LOC103707714, LOC103713163, LOC103697431, LOC103705017, LOC103722965 and LOC103722132) were related to calmodulin and calmodulin-like (CaM/CML) genes (Fig. 4A). The up-regulation of *CNGCs*, *Rboh* and *CDPK* indicated that Ca^2+^ plays very important role in signal transduction in date palm during infection. The expression of *nitric-oxide synthase* (C4H; LOC103710739) was down regulated in infected leaves as reported earlier in (Fig. 4A). We identified four DEGs: *mitogen-activated-protein kinase (MKK1*/2; *MKK1/3; MKK4*/5*)* and *WRKY transcription-factors* (*WRKY* 25/33) were found up-regulated in after infection (Fig. 4A). However, *WRKY22*/29 was found down-regulated during infection. Thus, the results suggesting the activation of pathogen-triggered immunity (PTI) pathways.

We have detected eleven genes related to effector triggered immunity (ETI) pathways during DB infection. Among these genes RPM1-interacting protein 4 (LOC103710585), *PRM1-interacting protein 4* (*RIN4*), two disease resistant associated genes (*SGT1*, LOC103708998; *HSP90*, LOC103722381), three R genes (*RPM1*, *PBS1*, and *RPS5*) and pto-interacting protein 1-like (LOC103707679; encoding serine-threonine kinase^36^) were found up-regulated. However, one *SGT1* gene was found down-regulated during infection. This suggests the participation of expressed genes in either HR or defense-response strategies, followed by cell-death (Fig. 4A). The DEGs expressed in healthy and infected samples associated with plant-pathogen interaction are shown in the heat map (Fig. 4B).

### Phytohormonal activation and transduction in Dubas bug infection

**Figure 5:**
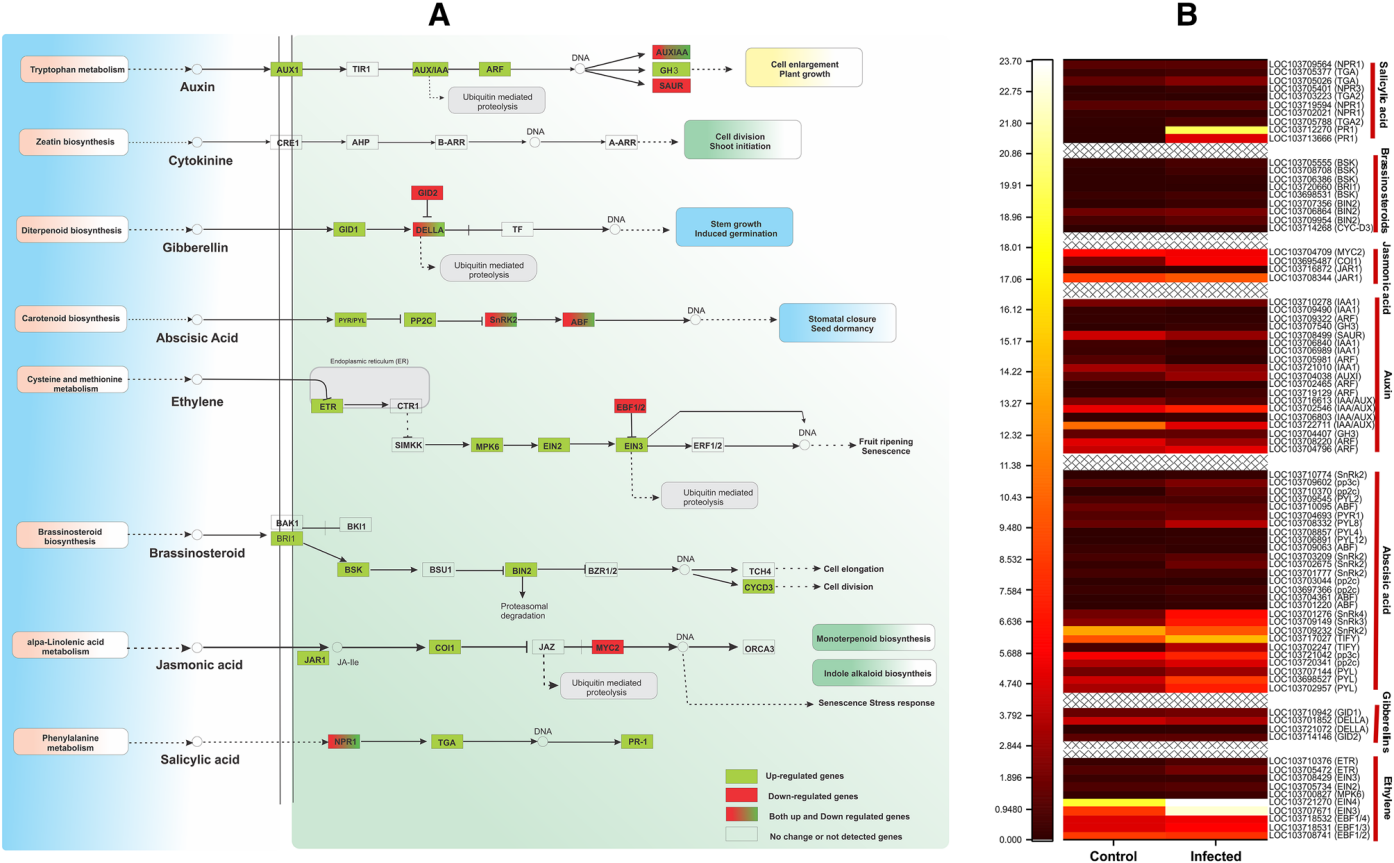
(**A**) DEGs involved in the plant hormone signal transduction pathway in response to Dubas bag infection enriched by KEGG analysis, (**B**) expression patterns of representative DEGs in plant hormone signal transduction pathway.

The results showed the presence of 85 DEGs associated with phytohormonal signal transduction pathways (Supplementary dataset 3). Auxin related genes were found up-regulated in infected samples suggesting activation of plant defense responses under DB attack. Eight out of 13 DEGs were up-regulated in infected samples and auxin-responsive protein *SAUR32-like* (LOC103708499) was found down-regulated as compared to control sample (Fig. 5A). Similarly, the jasmonic acid (JA) transduction pathway were up-regulated and two DEGs, *jasmonic acid-amido synthetase JAR1-like* (LOC103708344) and *coronatine-insensitive protein homolog 1b-like* (LOC103695487) were found upregulated whilst the transcription factor *MYC2-like* (LOC103704709) was found down-regulated in infected leaves as compared to control. The *NPR1* was found upregulated during infestation—a modulator SA signaling (Fig. 5A). Similarly, transcription factor *TGA2-like* (LOC103703223), which activate *pathogenesis-related protein PR1-2-like* (LOC103713666; LOC103712270) genes, showed similar expression and up-regulated after DB infection.

Further, DEGs associated with abscisic acid (ABA) related genes such as *abscisic acid receptors PYL* (LOC103702957; LOC103698527), *PYR* (LOC103704693), *probable protein phosphatase PP2C* (LOC103697366), *serine/threonine-protein kinase SAPK* (LOC103709232), and *abscisic acid-insensitive 5-like protein 5* (LOC103701220; LOC103704361) were up-regulated after infection as compared to control plants (Fig. 5B). This could suggest the involvement of ABA and SA—a possible resurrection of pathogenesis. In case of Brassinosteroid (BR), we found systemin receptor SR160-like (BR1:LOC103720660), *probable serine/threonine-protein kinase* (BSK; LOC103706386), *shaggy-related protein kinase eta* (BIN2; LOC103709954) and *cyclin-D3-2-like* (LOC103714268) were found up-regulated in infected leaves as compared to control (Fig. 5A). Ethylene (ET) related *ethylene receptor 2* (ETR; LOC103705472), *protein ethylene-insensitive 2* (EIN2; LOC103705734), *protein ethylene-insensitive 2* (EIN3; LOC103708429), and *mitogen-activated protein kinase 1-like* (MPK6; LOC103700827) were found up-regulated, whereas *EIN3-binding F-box protein 1-like* (EBF1/2; LOC103708741) was found down-regulated (Fig. 5AB).

### Secondary metabolites activation during Dubas bag infection for improved defense responses

**Figure 6:**
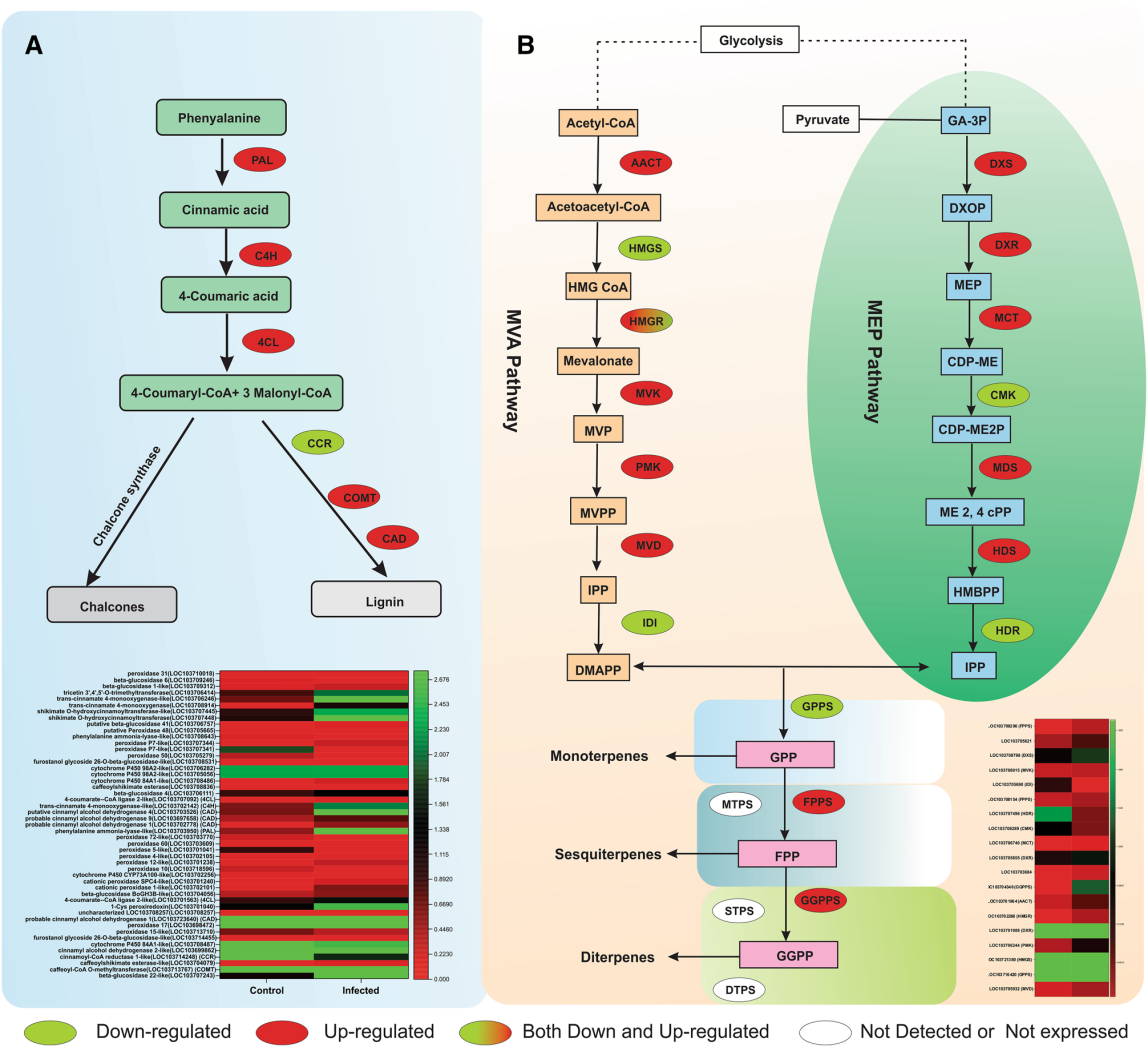
(**A**) Heat map and DEGs expression profiles of genes associated with phenylpropanoid biosynthetic pathway, (**B**) heat map showing the different expression profiles of genes involved in the terpene biosynthesis pathway. Adapted from Zulak and Bohmanu^37^, Liu et al.^38^. DEGs with an adjusted *p *value (*p*adj) < 0.05 were differentially expressed. AACT, acetyl-CoA acetyltransferase; HMGR, hydroxymethylglutaryl-CoA reductase; HMGS, hydroxymethylglutaryl-CoA synthase; MVK, mevalonate kinase; MVD, mevalonate diphosphate decarboxylase; PMK, phosphomevalonate kinase; DXS, 1-deoxy-d-xylulose 5-phosphate synthase; DXR, 1-deoxy-d-xylulose-5-phosphate reductoisomerase; CMK, 4-diphosphocytidyl-2-C-methyl-d-erythritol kinase; HDS, (E)-4-hydroxy-3-methylbut-2-enyl-diphosphate synthase; MCT, 2-C-methyl-d-erythritol 4-phosphate cytidylyltransferase; MDS, 2-C-methyl-d-erythritol 2,4-cyclodiphosphate synthase; IDI, isopentenyl di-phosphate isomerase; HDR, 4-hydroxy-3-methylbut-2-enyl diphosphate reductase; FPPS, farnesylpyrophosphate synthase; GGPPS, geranylgeranyl diphosphate synthase; GPPS, geranyl diphosphate synthase; DTPS, diterpene synthase; MTPS, monoterpene synthase; STPS, sesquiterpene synthase

In the biosynthesis of phenyl-propanoid pathway, up-regulation of genes that leads to synthesis of coumarinate from coumarin are also considered important in herbivory. Results showed that 38 DEGs out of 48 were up-regulated that were mainly associated with phenyl-propanoid pathway during DB infection (Fig. 6A). One of the key enzyme *phenylalanine ammonia-lyase-like* (PAL; LOC103703950) was up-regulated in infected samples. As DB attacks the leaf part by herbivore lignin, we found related genes viz. *cinnamyl alcohol dehydrogenase 1* (CAD; LOC103702778), *caffeoyl-CoA O-methyltransferase* (COMT; LOC103713767), *4-coumarate–CoA ligase 2-like* (4CL; LOC103701563), and *trans-cinnamate 4-monooxygenase-like* (C4H; LOC103702142) activated in infected samples compared with control (Fig. 6A).

Among secondary metabolites, we observed that DB infestation enhanced the mRNA levels of many genes associated with terpenoids and volatile compounds. Among 22 DEGs, eight were related to mevalonate pathways viz. *HMGR* (LOC103711741), *HMGS* (LOC103721380), *IDI* (LOC103705690) that were found down-regulated while *MVD* (LOC103705932), *PMK* (LOC103706244), *AACT* (LOC103701964) and *MVK* (LOC103708015) were found up-regulated in infected samples as compared to healthy samples (Fig. 6B). Similarly, in methylerythritol phosphate (MEP) pathway about seven DEGs such *HDS* (LOC103719733), *HDR* (LOC103713886), *DXR* (LOC103701988), *MCT* (LOC103706740), *CMK* (LOC103706289), and *DXS* (LOC103708798) were significantly expressed in DB infection leaves as compared to control (Fig. 6B).

**Figure 7:**
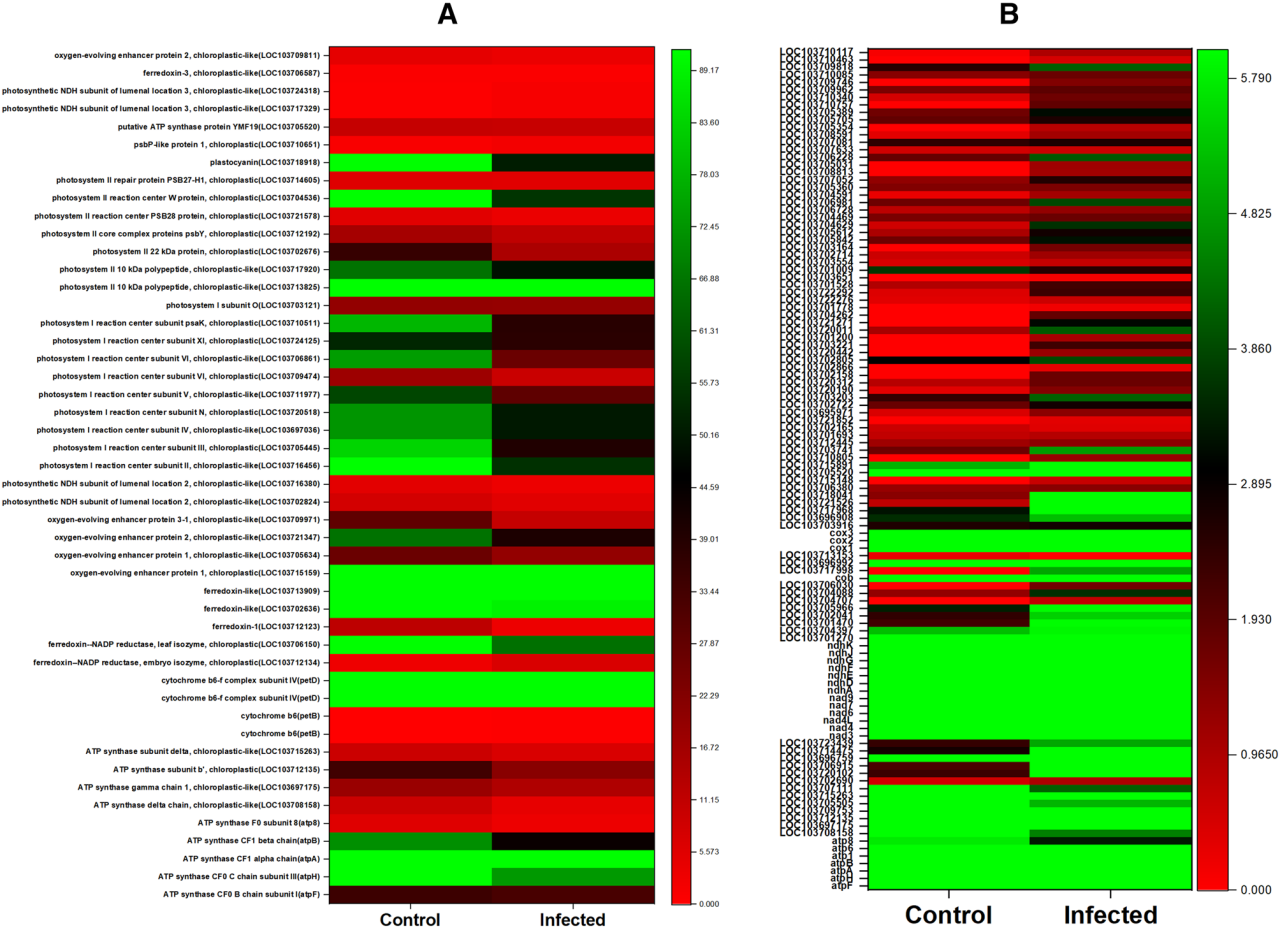
Expression patterns of representative DEGs in the oxidative phosphorylation (**A**) and photosynthesis (**B**). Heat map visualizing the expression patterns of the associated DEGs in Dubas bug infected and healthy date palm based on the FPKM value transcripts.

Results showed that genes involved in MVA pathways were relatively more expressed than MEP pathway. The results also showed a sole DEG related to IDI (LOC103705690) (Fig. 7A). Furthermore, the transcriptome data analysis revealed two DEGs related to GPPS and FPPS. The highly expressed DEG for FPPS (LOC103708154; LOC103709296) compared with GPPS also suggest a broader involvement of sesquiterpenes production in date palm. During DB infection, 48 genes were deferentially expressed in relation to photosynthesis process (Fig. 7A). In conjunction, about 110 DEGs were either up or down-regulated in response to oxidative stress metabolism for example *NADH dehydrogenase* (7 DEGs) and *NADH-ubiquinone oxidoreductase* (5 DEGs) during DB infection (Fig. 7B).

## Discussion

Studies have shown that herbivory induces oxidative stress by generating reactive oxygen species in infected leaf part^39,40^. Current results showed DB infection significantly increased O_2_^−^ as compared to control which is perceived as signaling molecules during plant innate immunity responses^41^. However, this oxidative burst could cause multitudinal damages, e.g. oxidation of lipid bi-layer^19^ especially by anionic oxides. Current results showed increased contents of malondialdehyde in infected leaf tissues suggesting a clear indication of increased peroxidation in the tissues^42,43^. To avoid such damages, plant recruit oxidative stress related enzymes such as superoxide dismutase (SOD), whereas it has been directly correlated with the production of O_2_^−^ and H_2_O_2_^19^. An increased level of SOD in DB infection predominantly suggest that date palm activates its antioxidant apparatus like other plants such as rice during herbivory^44^. We noted that mRNA gene expression of *PT2* and *NADPH-TR* were significantly activated during DB attack, suggesting their involvement in cell-wall modification and osmo-protectant activity^33,34^.

To counteract the negative impact of herbivory, phytohormones e.g. JA are identified as key signaling molecule to trigger defense responses in plants^17,45^. This has been well-attributed to pre and post herbivory attacks in plants^17–19^. Besides, SA has also been coined for its resilient role in enhancing resistance against insect induced pathogenic attacks^35^. The current results showed that JA and SA contents were not antagonistic to each other and both were higher as compared to control. This could be inter-correlated to the dual action that DB infection causes (a) herbivory and (b) pathogenesis. Since the process of dual antagonisms of such attacks could be highly complex, therefore, we performed transcriptome of infected and control seedlings to usher more molecular insights. Current study showed 10,042 DEGs, where among 69% (6,919) were up-regulated genes. GO annotation showed that 4,341 DEGs belonging to cellular, bio-chemical and molecular components. Recent study of Norway spruce tree’s transcriptome showed involvement of molecular function, biological process but no cellular component term during needle bladder rust, suggesting a variable responses of DB infection^46^. During DB infection, it was noted that 73 DEGs were related to plant-pathogen interaction, suggesting a post-herbivory genetic cascade.

In these interaction, calcium are considered vital for regulating plant responses to various pathogens and herbivory^47^. During such infection, the Ca^2+^ (calcium) signaling by CDPK and CaM/CML to produce ROS and NO separately, which induce defense responses^48^. We noted that CNGCs, CaM/CML and Rboh were upregulated and CDPK down regulated in DB infestation, suggesting the role of Ca^2+^ invasion^49^. Studies revealed that CNGCs are crucial in phytohormonal responses, pathogenesis/herbivory and plant immunity, by interacting with the ubiquitous Ca^2+^ sensor calmodulin as noted in Arabidopsis and rice^50–52^. CDPKs, on the other hand, modulate JA and ABA biosynthesis, plant stress tolerance, and plant fungal stimuli interaction because it is known as an important sensors of changes in Ca^2+^ levels^52,53^. The phosphatase enzyme, phosphorylate CDPKs which then play role in the hypersensitive response (HR). Previously, CaM/CML protein family regulate cellular responses to pathogenic induced HR responses in grapevine^54^. Activation of HR responses in DB infection could have played role in reducing infection caused by extricating cell death in neighboring cells^46^. A similar conclusion was also revealed by Trujillo-Moya et al.^46^ Norway spruce.

It was reported that during pathogen-associated molecular pattern (PAMPs)^55^, a considerable activation of MAPK and WRKY related genes are used to induce defense responses^36, 56^. These orthologues have been noted in current studies and are known to regulate pathogen-associated molecular pattern triggered-immunity (PTI) pathways. However, plants, in some cases can use effector trigger immunity (ETI) defense response strategy to cope with pathogenic attacks^36,56^. Both the pathways induce signaling cascades of ET, JA, and SA^57^ whereas, some of the studies also suggest the involvement of BRs and GA as well^58,59^ during infection process as was noted in case of Flg22 and BR biosynthesis^60,61^. Furthermore, auxin is associated with plant defense response^62^. During pathogenic fungi attack, plant activate defense signaling, in which auxin signaling role is well known, however it does not show this kind of relation under pathogens attack^63,64^. In the current study, we found that DEGs related to auxin biosynthesis were expressed significantly. We conclude that date palm can response to DB attack by modulating auxin, BR, GA, JA and ET signaling. Based on speculation that how several pathogen invades other plants^63^, we suggest that DB might secrete effectors into date palm tissues to improvise the infestation process.

Other than auxin, GA are regulated through DELLA protein family^69,70^ that also help to maintain SA and JA homeostasis ^65–67^ during infection process. Our findings are in synergy to Zhang et al.^68^ that these are up-regulated during fungal infection in grapes plants. Since, SA is involved in systemic acquired resistance (SAR) and regulation of plant defense responses^69^, we noted activation of *PR1* genes. SA is activate tolerance in plants against pathogenic attacks while ET and JA are centric against bio-necrotrophic pathogenesis^65,70, 71^. In addition to sole ABA, SA, and JA role, their antagonism such as SA vs JA has been reported in various plants^72^. We noticed that upon DB attack on date palm, most of upregulated DEGs were related to JA and SA signaling. The results suggest that the interaction of DB and date palm based on alternative processes as compared to that described in Arabidopsis and rice. Current study showed that DB has considerably novel infection process than other plants. Similarly, ABA also paly important role in plant experienced different biotic and abiotic stress conditions^69^. ABA trigger various expression of various genes responsible for physiological and developmental process in plants^73^. Likewise, several studies have reported the up-regulation of ABA under pathogen attack, suggesting its essentiality to reprogram JA dependent-defense responses^74^. Here, the increased ABA and SA level possibly indicates stress tolerance and SAR activities. In addition, BR confers pathogenesis related stress tolerance^60,75^, for example, exogenous BR substantially diminished the fungal-induced pathogenic attacks in potato^61,76^, whilst correlative impacts were also revealed in sugar beet, cucumber, rice and tomato etc^77^. Herein, we have found four up-regulated DEGs in the ET transduction pathways, suggesting preemptive role of ET in initial DB signaling during infection process^78^.

The phenyl propanoid biosynthesis pathway plays a vital role in plant physiology including plant-defense system by producing various chemical barriers against infection process^79^. Our results showed 38 upregulated DEGs that were associated with phenyl propanoid biosynthesis pathway during DB infection. Similar results were reported by Zhang et al.^68^ in grapevine under fungal (*Lasiodiplodia theobromae*) attack. Accumulation of these metabolites in infected samples suggest active contribution during plant-defense responses in date palm during DB infection. The metabolites of phenyl propanoid pathways leading into coumarinate was found in the current results. Coumarinate have been noted for their contribution in defense responses against phytopathogen, oxidative stresses and endogenous phytohormonal responses^80^. Chloropgenat and caffeate are phenolics, synthesized in the pathway with potential benefits in defense mechanism^79^ and scavenging ROS^81^. The results of the current study were consistent with previously reported study^82^. Similarly, increased expression of *4CL* and *PAL* genes in date palm are in synergy to grapevine after infection^68^. Moreover, similar results were recorded in Arabidopsis^83^. Hence, current results suggest that date palm can adopt *PB* pathways to regulate lignin synthesis and by activating secondary metabolites to hinder the DB infestation process.

In case of secondary metabolites, terpenoids are considered to play very important role against herbivores either directly or by attracting natural enemies of the attacking insects. Current results revealed 22 DEGs associated to terpenoid biosynthesis pathway were expressed significantly. We noted the expression of gossypol and its derivate that are known phytoalexin, lead to constitutive and inducible tolerance toward variety of pests^84^. Not only secondary metabolisms but primary metabolism was also active during DB infection. Numerous studies have demonstrated that herbivory can results in down-regulation of primary metabolic processes while simultaneously activating defense-related processes including secondary defense metabolism. As the result showed that DB infection showed 48 highly expressed genes, responsible for photosynthesis as compared to control. This indicate that date palm reactivate both primary and secondary metabolism under DB infection^85,86^.

## Conclusion

Dubas bug infection has been noted to enormously influence the endogenous plant innate immune responses, where a continued exposure leads to increased cell death. The results showed that the Dubas bug infection leads to the activation of endogenous JA and or SA initiating signaling cascades via the expression of 140 genes mostly belonging to cellular functions (calcium and MAPK), endogenous phytohormones (auxin, GA, ABA, ET, BR, JA and SA), and secondary metabolites (phenylpropanoids—coumarinates and mevalonate—gossypol). The hypersensitive responses of the leaves were significantly expressed during the infection process. This study performed for the first time detailed transcriptomic analysis of the infection and defense related responses of the date palm. These findings and the transcript dataset generated through this study would act as a future resource for qualitative trait loci of disease and insect resistance mechanisms in date palm.

## Material and methods

### Plant material, Dubas bug infection and RNA extraction

Healthy date palm (*Phoenix dactylifera* L., Khalas cultivar) seedlings were grown for one year in greenhouse conditions. The seedlings, in a randomized block design experiment, were used for two treatments (1) healthy control and (2) DB infected with each comprising of 30 seedlings. The disease infestation process was followed as mentioned previously^87^. To avoid dispersion of insect attack to neighboring plants or environment, the pots were shifted into environmental growth chamber (12 h of light at 30 °C (08:00–19:00; 12 h of dark at 30 °C 20:00–07:00; relative humidity 60%; Excelsior Scientific, UK) for rest of the experiment. The control seedlings were also grown at the same temperature conditions. After twenty days of infestation, leaf samples (three biological replicates) from each healthy control and infected seedlings were collected in liquid nitrogen for high molecular weight RNA extractions. RNA extraction buffer was prepared (Tris–HCl; 0.25 M, NaCl; 0.05 M, 20 mM; EDTA; (pH = 7.5); 1% (w/v) sodium dodecyl sulphate (SDS), 4% PVP w/v) as mentioned in the Liu et al.^88^. The quality of RNA was checked on formaldehyde agarose gel electrophoresis and quantified using Qubit 3.0 with dsRNA broad range kit (Thermo Fisher, USA). To remove the DNA contamination, the resulting RNA were treated with DNase I. The RNA was further checked on Bioanalyzer (Agilent, Germany), where a standard 7.0 to 7.5 RIN value was used to affirm suitability of RNA for onward NGS workflow and molecular analysis.

### Gene expression of infected sample by quantitative real time PCR

For cDNA synthesis, 1 µg RNA was used. High-Capacity cDNA Reverse Transcription Kit from ThermoFisher was used for cDNA synthesis. Specific conditions (25 °C for 10 min, 37 °C for 2 h and 85 °C for 5 min) of Polymerase Chain Reaction (PCR) was performed in thermo-cycler. The cDNA was stored in − 80 °C refrigerator. In healthy and disease samples, the expression of 4 genes (Table S1) were analyzed in replicate by qRT-PCR (QuantStudio 5.0 by Applied Biosystems Life Technologies), by using “SYBR” green Master Mix. The PCR reaction was carried out in triplicate. For all the primers, *Actin* gene was used is reference^42,89^. Threshold level of 0.1 was set for gene amplifications. Reaction conditions of qRT-PCR was following (94 °C for 10 min in stage 1, 35 cycles (94 °C for 45 s, 65 °C for 45 s, 72 °C for 1 min), with final extension at 72 °C for 10 min.

### Oxidative stress related parameter analysis of infected leaf with Dubas bug

The level of lipid peroxidation or formation of MDA was estimated using the methodology reported by Okaichi et al.^90^. Tissue homogenates were extracted with 10 mM phosphate buffer (pH 7.0). For the quantification of MDA, 0.2 mL of tissue homogenate was combined with 0.2 mL of 8.1% sodium dodecyl sulfate (SDS), 1.5 mL of 20% acetic acid (pH 3.5), and 1.5 mL of 0.81% thiobarbituric aqueous acid (TBA) solution in a reaction tube. Thereafter, the mixture was heated in boiling water for 60 min. After cooling to room temperature, 5 mL butanol: pyridine (15:1 v/v) solution was added. The upper organic layer was separated and the optical density of the resulting pink solution was recorded at 532 nm using a spectrophotometer. Tetramethoxypropane was used as an external standard.

The level of O2^·−^ was estimated using the method described by Gajewska and Skłodowska^91^. The homogenate for the reaction was prepared by immersing 1 g of fresh plant sample in 10 mM phosphate buffer (pH 7.0) , containing nitrobluetetrazolium (NBT) (0.05%; w/v), and sodium azide (NaN_3_) (10 mM), followed by incubation at room temperature for 1 h. Then, 5 mL of the mixture was taken in a new tube and heated for 15 min at 85 °C. Thereafter, the mixture was cooled and vacuum filtered. The absorbance was read at 580 nm with a spectrophotometer.

### Photosynthetic pigments determination

Chlorophyll and carotenoid contents were determined spectrophotometry according to Lichtenthaler^92^. Exactly 400 mg of fresh tissue of plant was mixed with 10 mL 80% acetone, the absorption was read with spectrophotometer at 663.2, 646.8 and 470 nm against acetone 80% blank. The concentration of chlorophyll (Chl) and carotenoid (Car) was determined by the following formulas:

$$\begin{aligned} {\text{Chl}}a\left( {\upmu {\text{g}}/{\text{mL}}} \right) & = {12}.{25}*{\text{A663}}.{2} - {2}.{79}*{\text{A646}}.{8} \\ {\text{Chl}}b\left( {\upmu {\text{g}}/{\text{mL}}} \right) & = {21}.{5}0*{\text{A646}}.{8} - {5}.{1}*{\text{A663}}.{2} \\ {\text{Chl}}t\left( {\upmu {\text{g}}/{\text{mL}}} \right) & = {\text{chl}}a + {\text{chl}}b \\ {\text{Car }}\left( {\upmu {\text{g}}/{\text{mL}}} \right) & = \left( {{1}000*{\text{A47}}0 - {1}.{8}*{\text{chl}}a - {85}.0{2}*{\text{chl}}b} \right)/{198} \\ \end{aligned}$$

### Phenolic acid and polyphenol quantification

The established protocol of the Zavala-López and García-Lara^93^, were used for the quantification of phenolic acid. Briefly, phenolic acid was extracted with 80% methanol. Exactly 0.7 mL were 80% methanol were used for 50 mg sample, after mixing the sample were vortex vigorously. Followed by incubation at 25 °C for 15 min, followed by centrifugation for 10 min at 5,000 rpm. The supernatant was decanted and stored at − 20 °C until analysis. The total phenolics were extracted and quantified using the improved extraction methods of Urias-Peraldí et al.^94^. To quantify total polyphenol, both healthy and disease sample (200 mg) were extracted with 80% ethanol. For assay, 100 µl extract was combined with 1 ml Folin–Ciocalteau reagent and 1 ml of 10% Na_2_CO_3_. After incubation for 1 h, tubes were centrifuge at 15,000 rpm for 10 min at 4 °C. The supernatant was taken and read at 760 nm in spectrophotometer. The Polyphenol content were expressed as mg/g.

### Endogenous phytohormonal analysis

The established protocol of McCloud and Baldwin^95^ and Khan et al.^42^ were used for the extraction and quantification of endogenous jasmonic acid (JA). Salicylic acid (SA) was extracted and quantified from freeze dried samples (control and infected) according to protocol of Seskar et al.^96^, as described by Khan et al.^97^.

### Transcriptomic sequencing of Dubas bug infected date palm

Looking at the initial results on the activation of endogenous hormones and antioxidants, transcriptome analysis was performed of infection and control samples to understand various underlying mechanistic pathways in date palm. Total RNA was extracted from the infected and control leaves collected (three biological replicates), using the Pure Link Plant RNA reagent kit (Life Technologies, USA) with some modifications to the extraction method. Quality, integrity and quantity of RNA were checked using gel electrophoresis and Qubit 3.0 fluorophotometer and Bioanalyzer (Agilent Technologies, Santa Clara, CA). The defined criterion for qualification of RNA for RNAseq is that the RIN value of a sample is higher than 7.6. The RiboMinus Plant Kit for RNA seq Kit (Invitrogen) was used to remove large ribosomal RNA (rRNA) from the total RNA, which was followed by the concentrating the rRNA depleted RNA using the RiboMinus concentration module, post-ribominus the RNA was evaluated on the Bioanalyzer (Agilent Technologies, Santa Clara, CA). The cDNA Libraries were constructed using the Ion total RNA-seq kit V2, ERCC RNA spike-in control mixes were added according to the manufacturer’s protocol using 100 ng of RNA. The total RNA was fragmented using RNase III followed by purification, and quantification on the Bioanalyzer. The resulting cDNA was amplified and quantified on the Bioanalyzer. The Ion One Touch 2 system (Life technologies USA) were used for the emulsion PCR of cDNA, which was followed by the Ion One Touch ES for the enrichment of template. The enriched template was loaded into Ion 540 Chips for the transcriptome sequencing on Ion Torrent S5.

### Sequencing and transcriptomic data analysis

The FastQC program (V 0.11.5), and Trimmomatic (V 0.36)^98^ were used for the assessment of Quality control, and adapter and poly-N contamination (low-quality sequences; phred ≤ 20) were removed. The remaining high-quality read were utilized for downstream analysis. From NCBI genome database, a reference genome and gene model annotation were downloaded (ftp://ftp.ncbi.nih.gov/genomes/Phoenix_dactylifera/GFF/). The Bowtie (v2.2.3) was used to developed index of the reference genome, and TopHat v2.0.12 used for paired-end reads were aligned to the reference genome Broad Institute), and number of reads mapped to each gene were determined by using the software HTSeq v0.6.1. The TopHat alignment results ware further investigate for construction and identification of both known and novel transcripts by using Cufflinks (v2.1.1) Reference Annotation Based Transcript (RABT) assembly approach^99,100^. RPKM (reads per kilobase of transcript per million mapped reads) and FPKM (fragments per kilobase of transcript per million mapped reads) were used in subsequent analyses^101^.

### GO ontology of DEGs

The Cuffdiff v.2.2.156 and DESeq R package v1.18.0 were used to identify DEGs in control (healthy) and infected treatments (Figure S3; Supplementary Dataset 1). The resulting *p* values were adjusted using Benjamini and Hochberg’s approach to control the false discovery rate (FDR)_ENREF_107^102^. Genes with an adjusted *p* value < 0.05 found by DESeq were assigned as differentially expressed. Most of the time, Gene Ontology (GO) analyses were conducted in order to investigate large-scale transcription data^103^. For the genetic studies, in order to determine the information related to the network among genes are usually determined by Kyoto Encyclopedia of Genes and Genomes (KEGG) pathway database^104^. In the current study, we used DAVID (version 6.8)^105^ to enrich the GO functions and pathways of specific DEGs in the KEGG (https://www.genome.ad.jp/kegg/) and GO (https://www.geneontology.org) databases and the R package 3.5.2 Goplot^106^ with an adjusted *p *value (q-value) of < 0.05.

### Supplementary information

Supplementary file1

Supplementary file2

Supplementary file3

Supplementary file4
