# Supplementary material for: Transcriptomic analysis of Dubas bug (Ommatissus lybicus Bergevin) infestation to Date Palm
Source: Sci Rep. 2020 Jul 13;10:11505. doi: 10.1038/s41598-020-67438-z (PMC7359322; doi:10.1038/s41598-020-67438-z)
Supplement: Supplementary file 1 — Supplementary file1 [file 41598_2020_67438_MOESM1_ESM.docx]

A Transcriptomic of Dubas bug (*Ommatissus lybicus* Bergevin) infestation to Date Palm

**Abdul Latif Khan^1#*^, Sajjad Asaf^1#^, Adil Khan^1#^, Arif Khan^1^, Mohammad Imran^2^, Ahmed Al-Harrasi^1*^, In-Jung Lee^2^, Ahmed Al-Rawahi^1^**

^1^Natural and Medical Sciences Research Center, University of Nizwa, Nizwa, Oman

^2^School of Applied Biosciences, Kyungpook National University, Daegu, South Korea

**Corresponding Authors:**

Abdul Latif Khan ([latifepm78@yahoo.co.uk](mailto:latifepm78@yahoo.co.uk)); Ahmed Al-Harrasi ([aharrasi@unizwa.edu.om)](mailto:aharrasi@unizwa.edu.om))

# Equally contributed to the article

**Supplementary Table 1.** The gene name, gene description, product size, reference number and oligonucleotide sequences used for qRT-PCR.

**Figure S1**. Date palm leaves infected by Dubas bug (DB) (A), Fold change expression of abscisic acid receptor PYL4-like expression. **, indicate a significant difference between healthy and infected sample where P ˂ 0.01.

**Figure S2**. Effect of DB infection on photosynthetic pigments **(A)**, Chlorophyll *a* **(B),** Chlorophyll *b* (C), Chlorophyll *a+b* (D), Carotenoids (E), Polyphenols, and (F) Phenolic acids. *, **, and ***, indicate a significant difference between healthy and infected sample where P ˂0.05, 0.01, and 0.001 respectively. While ns indicate non-significant difference, by using two-way ANOVA.

**Figure S3**. Healthy and infected data analysis and mapping to reference genome. (A) density of DEGs in the data-set, (B, C) volcano and scattered plots of DEGs in data-set by cummerbund (CuffDiff), (D) a dendrogram base on mapped read counts was constructed for the four analyzed samples belonging to healthy and infected (Dubas bugs), (E, F, G, H), gene dispersion, top hundred (100) significantly expressed DEGs heatmap and PCA plots of DEGs analyzed by DESeq2 in the data set.

**Supplementary dataset 1.** Complete list of DEG detected in this study in healthy (control) and infected plants.

**Supplementary dataset 2.** DEGs involved in plant -pathogen interaction pathway in date palm infected by Dubas bug based on KEGG analysis.

**Supplementary dataset 3.** Genes involved in the plant hormone signal transduction pathway.

**Supplementary Table 1.** The gene name, gene description, product size, reference number and oligonucleotide sequences used for qRT-PCR

**Figure S1**.

**Figure S2**.

**
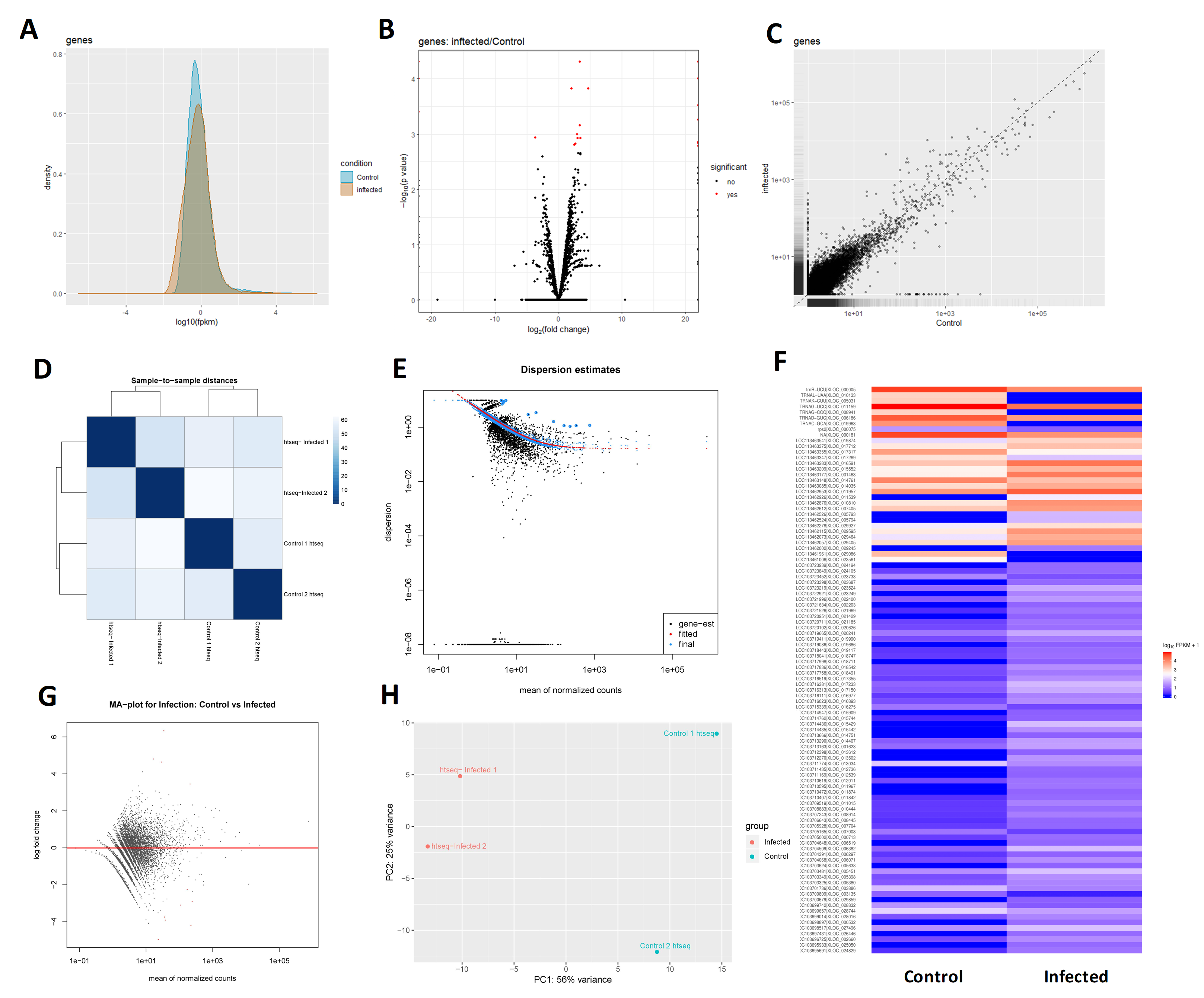
**

**Figure S3**.
